# Field observation as a method to guide patient-reported outcome measurement integration in community cancer centers

**DOI:** 10.1186/s43058-026-00859-5

**Published:** 2026-01-28

**Authors:** Manraj N. Kaur, Amanda Higgins, Maria O. Edelen, Kelly A. Aschbrenner, Meghan E. Garstka, Alyssa Throckmorton, Vivian Bea, Andrea L. Pusic

**Affiliations:** 1 Patient-Reported Outcomes and Values, & Experience Center (PROVE), Department of Surgery, Brigham and Women’s Hospital, Harvard Medical School, 75 Francis Street, Boston, MA 02115 USA; 2 Geisel School of Medicine at Dartmouth College, 1 Rope Ferry Rd, Hanover, NH 03755 USA; 3 Breast Surgical Oncology, University of Maryland Medical System, 22 S. Greene Street, Baltimore, MD 21201 USA; 4 Baptist Medical Group, Memphis Breast Care, 7205 Wolf River BLVD STE 200, Germantown, TN 38177 USA; 5 Department of Surgery for NewYork - Presbyterian, Brooklyn Methodist Hospital, 506 Sixth Street, Brooklyn, NY 11215 USA

**Keywords:** Patient-reported outcomes, Breast cancer, Implementation, Field work, Site visits, CIFR, Determinants

## Abstract

**Background:**

Field observation is a valuable but underused methodological approach in patient-reported outcomes (PRO) implementation research, particularly in low-resourced settings such as community cancer centers (CCCs). Rooted in ethnographic tradition, field observations allow researchers to assess clinical environments in real time, capturing workflow processes, communication patterns, and contextual factors not readily accessible through interviews or surveys. When applied through implementation science frameworks such as the Consolidated Framework for Implementation Research (CFIR), this method supports systematic assessment of organizational structures, implementation climate, and readiness for change. The objective of this study was to develop and apply a structured field observation protocol to inform context-specific PRO implementation workflows in CCCs providing breast cancer care.

**Methods:**

Structured field observations were conducted at five CCCs during the pre-implementation phase of a larger initiative to integrate imPROVE, a web-based PRO data collection platform, into routine care. The protocol followed three phases: (1) pre-visit planning with site leads to gather contextual and logistical data; (2) in-clinic observations by two trained researchers documenting clinic layout, patient flow, staff roles, and communication; and (3) post-visit data processing using structured templates and CFIR-guided analysis. Field notes were triangulated with insights from staff, patient and community leader interviews (reported elsewhere) to generate site-specific implementation strategies.

**Results:**

Observations uncovered key contextual differences across sites, including clinic layout, staffing stability, patient volume, caregiver presence, and digital literacy. These variations influenced feasibility and shaped tailored implementation plans. For example, sites with long waiting periods between check-in and exam were better suited for waiting-room PRO collection, while others required provider-facilitated approaches. Language diversity and caregiver engagement also emerged as critical determinants. Sites with strong leadership continuity and clear workflows demonstrated higher implementation readiness.

**Conclusion:**

Field observation was instrumental in identifying real-world barriers and facilitators that shaped site-specific PRO workflows. This approach enhanced ecological validity, stakeholder engagement, and the feasibility of PRO integration in CCCs. Embedding field observation in early implementation planning strengthens the methodological rigor of PRO research and supports sustainable, context-aligned interventions in resource-constrained settings.

**Supplementary Information:**

The online version contains supplementary material available at 10.1186/s43058-026-00859-5.

Contributions to the literature

- Pre-implementation field observations are rarely used in PRO implementation studies; this study shows how they can identify practical barriers and opportunities before launch.
- We provide a detailed, structured framework for pre-implementation field observation and templates for assessing workflow at clinical settings, making it easier to assess local readiness and tailor implementation plans.
- Our findings show how even well-resourced cancer centers vary widely in workflows and staffing, highlighting the need for flexible, context-specific implementation strategies.

## Background

Field observations represent a critical methodological tool that can be used to facilitate patient-reported outcomes (PRO) implementation research, offering a sophisticated approach to understanding complex healthcare environments through naturalistic inquiry [1]. Field notes provide rich, descriptive, and multi-faceted accounts of study environments, interpersonal interactions, and contextual determinants that shape PRO implementation, delivering insights that are difficult to capture through interviews or surveys alone [2]. Although long established within sociological ethnography and anthropology [3–5], field observations remain underutilized in PRO implementation science, representing a significant opportunity for methodological advancement. When guided by implementation science frameworks such as the Consolidated Framework for Implementation Research (CFIR), field observations can systematically document organizational structures, leadership engagement, culture and workflow dynamics – core components of the individuals and inner setting domain that influence PRO adoption and use [6, 7]. They also allow real-time assessment of how PRO data collection processes align with existing clinical workflows, supporting analysis of intervention characteristics, such as complexity, adaptability and relative advantage [6, 7].

When incorporated early in the implementation process, field observations capture contextual determinants critical for tailoring strategies to local needs [8]. Their high ecological validity ensures fidelity to real-world clinical practice conditions [9], enabling both qualitative and quantitative data capture that offer a comprehensive view of implementation environment. Embedding field observations into PRO implementation enhances rigor, trustworthiness, and readiness assessment [2, 10], strengthens understanding of implementation climate [11, 12], and supports the development of adaptive, contextually aligned PRO implementation models suitable for diverse healthcare settings. When triangulated with interview and survey data, insights from field observations further bolsters stakeholder engagement, feasibility assessment, and sustainability. However, despite these strengths, guidance on systematically incorporating field observations into PRO implementation research remains limited.

Non-PRO implementation research offers compelling evidence of what PRO implementation science currently lacks. A substantial body of work demonstrates that ethnographic and observational methods are uniquely effective for capturing context, revealing workflow patterns, communication dynamics, and latent barriers that structured assessments often miss [13–18]. A recent review by Gertner et al. illustrates how ethnography has been used to assess determinants of implementation, evaluate strategies, and generate new methodological tools, with observations elucidating decision-making processes and contextual constraints that shaped implementation trajectories [14]. Another scoping review extends this perspective through analysis of 227 implementation studies, documenting widespread use of observational and ethnographic approaches, including field journals, to characterize implementation processes, describe interactions between interventions and local environments, and identify multilevel determinants across healthcare and community settings [19]. Taken together, these bodies of evidence underscore the consistency with which ethnographic methods expose nuanced contextual determinants essential for strategy design, highlighting a clear and actionable opportunity for PRO implementation science to leverage these established techniques more systematically.

This study applies field observations at five community cancer centers (CCCs) as the foundation for developing tailored PRO implementation workflows for women receiving breast cancer care. The CCC site visits documented clinic layouts, workflow structures, and opportunities for integrating “imPROVE”, a breast cancer care-specific PRO web application, into routine practice [20]. Findings from these observations, combined with in-depth interviews with clinic staff, patients, and community leaders (not the focus of this manuscript) informed CCC-specific PRO implementation workflows. The primary objective of this paper is to present a comprehensive, structured framework for collecting, analyzing, and disseminating field observation data, expanding the methodological toolkit available to PRO implementation scientists and supporting more nuanced, context-responsive, sustainable PRO integration.

## Methods

This study represents the preparatory phase of a broader research program aimed at developing and implementing routine, health information technology (HIT)-assisted PRO data collection using imPROVE within CCCs serving patients with breast cancer. CCCs were identified through coordinated outreach by the Association of Cancer Care Centers (ACCC) and targeted networking with breast cancer surgeons. From the interested sites, five CCCs were selected based on diversity of patient served and their demonstrated capacity to support the planned implementation activities. The goal of the research program is to enhance access to the benefits of PRO reporting for patients seeking breast cancer care in CCCs.

Human research ethics approval for the site visits was obtained from the Dana Farber Cancer Institute’s Research Board (IRB 23–558). Formal written consent from clinic staff was not required because observations focused on workflow and system-level processes rather than individual performance. No Memorandum of Understanding was required because the partnering CCCs already had institutional collaboration pathways with the lead investigative team; however, all sites provided administrative authorization for the observational visit as part of project onboarding. The site visit protocol was structured into three distinct phases: pre-visit preparation, during-visit observations, and post-visit data processing and analysis. Each phase was carefully designed to ensure the data collected could directly inform the PRO implementation workflow strategy.

### Pre-visit preparation

The pre-visit preparation phase employed a structured, multi-step approach focused on contextual assessment and stakeholder engagement, allowing systematic information gathering about the CCC. The research team first established close collaboration with the site’s lead investigator and site champion to identify key administrative contacts (e.g., clinic manager) essential for visit planning. Research team members conducted at least one pre-visit telephone or video conference with administrative staff to collect contextual data including clinic operational hours, optimal observation days (coinciding with the clinic's busiest day of the week to ensure that field observations were indicative of a full clinic day), clinic location, and anticipated staff presence during the scheduled visit. Observers also gathered information regarding available workspace for the research team within the clinic setting and clarified institutional requirements such as occupational health and/or security clearances. During these preliminary interactions, the research team communicated the site visit objectives and provided a structured overview of the visit protocol. Structured observation templates used during visits were developed in-house based on anticipated determinants relevant to HIT-enabled PRO implementation in low-resource clinic settings and were iteratively refined across site visits to ensure adequate capture of workflow, environmental, and staffing determinants. When feasible, follow-up communication was scheduled to confirm logistical details, with final visit parameters documented through email correspondence for fidelity. The site visit protocol included strategic communication timing, with formal reminders distributed to both administrative personnel and the site’s lead investigator and champion one week and one day before the scheduled visit. This approach reflects implementation facilitation techniques emphasizing proactive planning, stakeholder preparation, and relationship-building [21]. Observations were conducted independently from interviews; interviews occurred after site visits to avoid disrupting clinic operations and to maintain the integrity of naturalistic workflow observations. The observers were trained in implementation science methods and had extensive experience with clinic workflow assessments and PRO implementation processes. Effective observation required familiarity with determinants of implementation (i.e., CFIR), ability to recognize workflow adaptations, and strong interpersonal skills to build rapport while remaining unobtrusive. This systematic pre-implementation assessment assisted the research team with developing a contextually tailored field observation strategy. By conducting thorough pre-visit information gathering, the research team enhanced site visit readiness, identified potential determinants of successful site visits, orchestrated strong communication and collaboration with site personnel, and established the relational foundation necessary for effective observational data collection in complex CCC settings.

### During the visit

The field observation phase focused on capturing clinic context and workflow dynamics while establishing rapport with the clinical staff. Both observers entered the visit with established rapport through prior meetings with site champions, clinical staff, and program administrators, ensuring shared understanding of study goals and facilitating trust during observation. Each observation spanned one full clinic day (approximately 7–8 hours), scheduled on the site's busiest clinic day. Two members of the research team arrived at least 15 min before clinic operations commenced to conduct preliminary orientation. Administrative or clinical personnel facilitated clinic orientation and introduced the team to key clinical stakeholders. The observers employed a strategic observational approach to document the implementation context. They conducted a systematic assessment of the physical environment (including patient-facing materials such as brochures and informational displays) and focused on front desk-patient interactions and interprofessional communication patterns. The observers did not directly observe patient appointments but systematically documented provider-patient interaction durations. Using strategic positioning, one observer was stationed at the front desk area to document patient flow processes including arrival timing, check-in procedures, and transition patterns to examination areas. This observer recorded caregiver presence, assessed front desk workload capacity, and documented additional administrative responsibilities that might influence implementation. The second observer, positioned in the clinical back area, documented patient rooming timing and provider interaction durations. This observer distinguished between new and follow-up patient appointments, assessed clinical staff capacity constraints, and observed and documented implementation climate factors such as staff attitudes towards PRO integration in clinic workflow, how effectively information flowed between administrative staff, providers and patients, and physical environmental constraints (layout limitations, space for patients to complete PROs). Informal conversational exchanges with clinical and administrative staff occurred naturally and were included as contextual insights within handwritten field notes. No audio or video recordings were used, and photographs of clinic spaces and materials were taken only prior to patient arrival and without identifiable individuals. Whenever possible, both observers fostered open communication with staff members throughout the day and collected additional implementation determinants including patient language diversity, appointment adherence patterns, reasons for missed appointments, and prior research experiences including PRO data collection, observable indicators of staff burnout, use of temporary or floater staff, and staffing consistency. The research team provided a catered, shared meal for clinical staff to acknowledge their participation and foster collaborative relationships essential for implementation success.

### After the visit

The post-implementation assessment phase comprised three components: (1) post-visit debriefing; (2) data management; and (3) structured data analysis.

#### Post-visit debriefing

Immediately following the site visit, the two field observers engaged in deliberate debrief session to document initial impressions, emerging determinants, and potential workflow adaptions relevant to PRO integration. While observation templates emphasized workflow and structural determinants, informal conversations and reflexive memos allowed the observers to capture cultural and relational dynamics that surfaced during the visit. Additional members of the broader study team (clinicians, health service researchers, epidemiologists, implementation scientists) contributed to interpretive discussions when appropriate. This reflective practice enabled identification of affective tone, non-verbal cues, and unanticipated contextual factors that would inform subsequent visits and refinement of the observation approach.

#### Data management

Handwritten field notes, photographs of clinic spaces, and structured observation templates were entered into an institutionally approved digital platform (Microsoft OneDrive). No identifiable staff information was retained. All data were organized into predefined observation templates to construct site-specific contextual profiles. Additionally, the direct costs associated with conducting site visits were tracked at an itemized level for each observer (e.g., travel, lodging, meals, transportation, supplies).

#### Data analysis

Observation templates were intentionally aligned with CFIR domains and constructs, enabling direct linkage between data collection and analysis. The team collaboratively applied CFIR's constructs to characterize determinants associated with integrating imPROVE into CCC workflows and identify potential barriers, facilitators and necessary workflow modifications. Following data organization, field observation summaries and insights were organized into factors influencing the integration of the imPROVE PRO data collection platform. Field observation summaries were shared with site leadership (site lead investigator and site champion) and their designated clinical or administrative representatives for refinement and validation, an approach consistent with best practices in implementation partnership development [21].

## Results

The site observation component of the implementation study was conducted at five CCCs January – May 2024. Four CCCs required single-clinic assessments, while one site (Site 1) necessitated two separate clinic observations due to scheduling constraints. Mean expenditure of $2,923 per observer, encompassing transportation (flights, airport transfers, site-to-hotel transport), accommodations and meals and stakeholder engagement provisions (lunch and refreshments for CCC staff) were incurred.

The pre-visit requirements exhibited substantial heterogeneity across the CCCs. One site (Site 1) mandated extensive non-patient-facing clearance procedures administered through occupational health services. These requirements included comprehensive immunization documentation and current vaccination verification (influenza and COVID-19). This implementation site also required the completion of multiple institutional forms: observer documentation, information systems confidentiality agreements, criminal background acknowledgment, and HIPAA privacy certification. The site access protocols varied significantly at other sites, with one requiring security check-in procedures and temporary visitor credentialing, while the remaining three sites did not enforce any formal documentation requirements for research team access.

### Innovation (intervention) characteristics

CCC site observation revealed significant heterogeneity in contextual factors likely to influence PRO data collection integration (Table 1). The adaptability of the imPROVE platform was a critical consideration for its successful integration across diverse CCCs. Site 1, with its substantial linguistic diversity, including English, Spanish, and Haitian Creole, would require multilingual platform capabilities to ensure equitable patient engagement. In contrast, Sites 2 and 3 serve predominantly English- and Spanish-speaking populations, reducing the complexity of language adaptation but still necessitating bilingual support materials. These linguistic variations highlight the need for the PRO collection platform to accommodate diverse patient populations, while maintaining implementation fidelity.

**Table 1:** Community cancer center description – for breast cancer surgery

Characteristic	SITE 1	SITE 2	SITE 3	SITE 4—Location 1	Site 4—Location 2	Site 5
**Clinic hours**	9:00 am-4:00 pm	8:00 am – 4:30 pm	8:30 am-3:30 pm	9:00 am-12:00 pm	1:00 pm-3:30 pm	9:00 am-4:00 pm
**Clinic days**	Mon-Fri	Mon-Thur	Mon, Tues, Fri	Mon-Fri	Mon-Fri	Mon & Thur
**Average patient volume**	45–70 patients/day across all providers	~ 30 currently, ~ 55 when fully staffed	~ 20/day per surgeon	~ 15–20/day, 20% of Multi-D	~ 20/day	New diagnoses: 5/week Follow-ups: 10–15/week
**Primary Languages—Patients**	English, Spanish, Haitian Creole	English, Spanish	English, Spanish	English, Spanish		English, Spanish, Haitian Creole
				English, Spanish		
**Staff-identified challenges**	Older population	Information overload for new patients	Most patients 55–75 years old	E-literacy, elderly	Hard to track, shared space with many other clinics	Most patients 55–75 years old
**Inforation sent to patient prior to clinic visit**	No - new patients get a printed packet on check-in	Yes – new patients receive an email	No – actively trying to transition to MyChart but often miss to send/patients do not access	Yes, through the patient portal – not always filled out		No, used to send one but discontinued
**No shows**	Very common, 2–3 per clinic day	20% - most of whom are scheduled with the nurse practitioner. The most common reason given by patients is lack of transportation	Minimal	Moderate - cause multifactorial		Minimal
**Staff research burden**	Minimal research burnout; staff workload burnout exists	N/A—well staffed. Numerous ongoing research projects	None to Minimal	Moderate		Minimal – Social Worker collects PHQ9 for research (paper); Research Nurse available
**Patient research burden**	Minimal	Minimal	Minimal	Potentially high – offered participation in a lot of research projects across the institute		Minimal
**Other details**	-	Two surgeons left the organization in early 2024 creating additional burden on existing providers. The new surgeon started in July 2025 Added another Nurse Practitioner and Physician Assistant in June 2024.	Many patients arrive early; paperwork is completed in the waiting room and then uploaded to the chart by the front desk	Shared space with busy breast imaging; imPROVE not feasible in the waiting room	Shared clinic space and check-in staff with 3 other clinics ; imPROVE not feasible in the waiting room	High proportion of Medicaid patients; Social Worker organizes month -support groups with 6–7 attendees (survivors and newly diagnosed) that last approximately 1 hr and is an opportunity to inform re. PRO data collection program
**Study research assistant identified at the time of the visit**	No	Yes	Yes	No	No	No, dedicated scribe S helps with research

The complexity of integrating imPROVE into existing workflows also differed across sites. Sites 2 and 4 have established patient portal systems to support pre-visit intake distribution, potentially enabling smoother integration pathway. However, inconsistent portal uptake poses challenges for standardizing pre-visit engagement strategies. Sites 1, 3, and 5 do not employ structured pre-visit information distribution, requiring alternative engagement approaches such as in-clinic education or clinician-driven PRO introduction.

### Outer setting

This domain captured findings related to system-level external pressures, incentives, and community-level factors. Across all CCCs, broader contextual influences shaped PRO implementation feasibility. Sites serving socioeconomically diverse catchment areas described substantial structural barriers to patient technology engagement, ranging from variability in device access to challenges navigating digital platforms. These community-level contextual factors influenced staff expectations regarding imPROVE uptake and the anticipated level of patient support required at each site.

### Inner setting

Significant heterogeneity in site characteristics was observed. Patient volume varied across sites, with Site 1 demonstrating the highest daily patient load (45–70 patients), followed by Sites 2 and 3 (20–30 patients). Sites 4 and 5 had lower volumes. The observed patient counts across front-end and back-end workflow assessments reflect these variations, with Site 1 documenting the highest number of patient observations (45 front-end, 38 back-end), whereas Site 4 reported the lowest (6 front-end, 6 back-end across two locations) (Table 1).

Structural characteristics further shaped implementation feasibility. Differences in clinic layouts and shared spaces influenced the practicality of waiting room-based PRO collection. Site 4’s shared waiting areas across multiple clinics and specialties presented logistical challenges, whereas sites with dedicated oncology clinic waiting areas may offer more predictable opportunities for PRO data collection.

Staff configuration and role distribution within CCCs demonstrated significant heterogeneity (Table 2). Medical assistant staffing ranged from one to three personnel per site, with Site 4 employing a floating coverage model during high-volume periods. Front desk staffing ranged from one to three personnel, with Site 2 demonstrating the most structured delineation of responsibilities between scheduling and in-clinic patient management. Leadership engagement varied as well. Site 1 experienced a transition in the lead investigator post-visit, temporarily affecting continuity in implementation planning. Site 2 was short two breast surgeons. Patient navigators, who could serve as key facilitators for patient engagement, were present at Sites 3 and 5 only. Similarly, social work support, which may be critical for managing distress screening outcomes within PRO collection, was only available at Sites 1, 2, and 5. Site 5 uniquely employed a medical scribe, presenting an opportunity for streamlined documentation integration not available at other sites. Surgery scheduling roles also differed substantially, with Site 2 maintaining external schedulers based at affiliated imaging centers. These findings reflect substantial variability in readiness and available human resources for PRO integration.

**Table 2:** Healthcare team description at the five community cancer centers – breast cancer surgery

	**SITE 1**		**SITE 2**		**SITE 3**		**SITE 4 – LOCATION 1**		**SITE 4 – LOCATION 2**		**SITE 5**	
STAFF	During visit	Part of team	During visit	Part of team	During visit	Part of team	During visit	Part of team	During visit	Part of team	During visit	Part of team
**Breast surgeon**	1	2	1	2	2	2	1	2	1	2	1	1
**Nurse Practitioner**	-	-	1	3	0	0	1	1	1	1	-	2
**Physician Assistant**	2	2	-	2	-	-	-	-	-	-	-	-
**Registered Nurse**	-	-	1	1	-	-	-	2	-	-	-	1
**Medical Assistant**	2	3	3	3	2 (1 in training)	2	1	1	1 *floater present on busier days	1 *floater present on busier days	2	2
**Front desk staff**	1	1	3	3	1	1	3	3	2	2	2	2
**Schedulers/access nurse**	1	1	2	2	1 *same as the front desk	1 *same as the front desk	5	5	-	2	1	1
**Nurse Navigator**	-	-	2 *also the RNs	2 *also the RNs	1	1		2	-	1	1	1
**Social Worker**	-	1	1 *also one of the RNs	1, also one of the RNs	-	-	-	-	-	-	1	1
**Scribe**	-	-	-	-	-	-	-	-	-	-	1	1
**Surgery scheduler**	1	1	2	2	2 *same as MA	2 same as MA		1	-	1	1	1
**Patient Navigators**	-	-	-	-	2	2	-	-	-	-	1	1

### Individuals (patients, caregivers, clinicians)

Patient needs and resources emerged as central determinants of implementation. Most CCCs primarily served older populations (ages 55–75 years), a group for whom digital literacy was frequently cited as a potential barrier. This influenced the anticipated level of in-person facilitators required for PRO completion. The proportion of patients arriving with caregiver varied from 10 to 50%. At sites with higher caregiver presence (e.g., Site 3), caregivers represented an additional support mechanism that could be leveraged for PRO engagement. Clinician-level determinants also emerged. Providers expressed varying degrees of comfort with technology-enabled workflows and differed in their expectations for staff involvement in PRO administration. Staff attitudes toward PRO relevance and anticipated workflow impact further shaped perceived feasibility.

### Process

The timing and placement of PRO data collection within clinic workflows emerged as a critical implementation consideration. Clinic flow analysis demonstrated substantial variation in appointment duration, ranging from 15-min follow-up visits to 180-min multidisciplinary evaluations (Table 3). The transition between front desk check-in and exam-room placement consistently presented the most viable PRO completion window, ranging from 4 to 65 min. Sites with consistently longer transition times (e.g., Site 5) may be suited for waiting-room-based PRO engagement, whereas sites with limited wait times may require alternative strategies, such as provider-initiated PRO completion or tablet-assisted entry during clinical encounter. The number of patients observed at each site highlighted workflow variability, reaffirming the need to tailor imPROVE integration strategies to the specific appointment structures, staff availability, and patient needs observed at each CCC.

**Table 3:** Average time spent by patients observed in community cancer centers – breast cancer surgery

	**SITE 1**		**SITE 2**		**SITE 3**		**SITE 4 (both locations)**		**SITE 5**	
	New	Follow-up	New	Follow-up	New	Follow-up	New	Follow-up	New	Follow-up
Patient arrival to check-in	0–8	0–8	0	0	0	0	2–5	2–5	0	0
Check in to the exam area	5–15	5–15	4–30	4–10	5–20	5–60	10–23	10–23	35–65	35–45
Exam area to clinic room	5–15	5–15	0–2	0–2	0–2	0–2	0–2	0–2	1–2	0–2
With medical assistant	5	5	2–5	2–5	5	3–5	2–12	2–5	5–10	5
With nurse practitioner/physician assistant	15–30	15–30	15–30	5–10	15–25	-	30–45	10–15	20–25	15 (w Surgeon)
With breast surgeon	60	15	30–45	5–10	25–50	15–25	15–20 (with MA)	10–15 (may not see breast surgeon)	25–30	10–15
Total time patient in clinic	90–130	45–90	50–110	15–40	45–100	30–90	120–180^a^	35–60	120–150^a^	65–80
							60–110^b^		90–130^b^	
% arriving with caregiver	10%	10%	25%	10%	50%	50%	20%			33%

^a^for multidisciplinary clinics

^b^for breast cancer surgeons only

Across all CFIR domains, interventions, outer setting, inner setting, individuals and process, the results consistently demonstrate that substantial site-level variability shapes PRO implementation feasibility. The field observations collectively indicate the need for tailored, site-specific implementation strategies that preserve core imPROVE functions while adapting workflow, staffing, and patient-engagement approaches to local contextual conditions.

## Discussion

This study reports on field observation at five CCCs before the implementation of a HIT-enabled PRO data collection program. As a methodological contribution, it demonstrates how structured field observations can function as a foundational PRO implementation planning tool. The marked heterogeneity across CCCs underscores the need for approaches that are both flexible yet structured, enabling strategy tailoring to local clinical environments while leveraging existing workflow patterns to support PRO engagement. Field observations provided insight into contextual elements that are difficult to capture through interviews or surveys alone, including workflow friction points, tacit norms in staff communication, and informal role dependencies, that directly share daily clinic operations. Staff reliance on unspoken verbal cues, redistribution of tasks during high volume periods, and improvised clinician flow in constrained physical spaces exemplify how routine, taken-for-granted behaviours shape implementation feasibility. These observations also revealed relational dynamics, such as interprofessional communication patterns and subtle hierarchies that influence workflow decisions. Together, these tacit and cultural practices offered essential information for identifying feasible integration points for PRO workflows at low-resourced settings.

Through the operationalization of CFIR domains within structured observational protocols, this study demonstrates how implementation determinants can be systematically documented during the pre-implementation phase using the principles of naturalistic inquiry. Naturalistic inquiry is a qualitative approach focused on prolonged engagement, holistic attention to context, and the value of observing real-world practices rather than eliciting post hoc accounts [22]. Naturalistic inquiry serves as the epistemological foundation for this study due to its alignment with implementation science’s emphasis on understanding complex systems through in-situ observation and iterative sensemaking.

The heterogeneity in implementation contexts documented across our five CCCs illustrates Weiner's organizational readiness theory in practice, demonstrating that clinics sharing similar mission can differ substantially in implementation capacity due to underlying structural and process distinctions [23]. Our findings also align with Greenhalgh’s diffusion of innovation framework, highlighting value of early identification of structural compatibility challenges to prevent misalignment between intervention design and real-world workflows [24]. Variability in pre-visit processes and waiting periods, for example, emerged as both an opportunity (natural intervention point) and a challenge (potential implementation barriers requiring tailored facilitation strategies).

A major advantage of structured field observation is its capacity to reveal tacit operational norms that influence implementation feasibility. Organizational differences, such as staffing stability, cross-coverage expectations, and informal task allocation, had direct implications for how implementation outcomes such as fidelity, feasibility, or adoption could be measured across sites. Observations equipped facilitators with contextual knowledge that reduces information asymmetry when entering new clinical environments [25–27]. Documenting both explicit organizational characteristics and tacit practices supports more accurate matching of strategies to context. These findings also reinforce arguments for prioritizing fidelity to function rather than form when implementing interventions across heterogeneous settings [28, 29]. Standardized implementation outcomes must account for contextual variation, potentially requiring site-specific benchmarks or adapted metrics that reflect differences in clinical workflows, staffing structures, and patient populations. Implementation outcomes should be framed within a context-strategy-outcome fit model, ensuring that variation in local determinants is incorporated into evaluation frameworks [30].

To support future research and practice, we developed a field observation framework for PRO implementation, informed by our team’s field experience and post-visit discussions. This framework, presented in Table 4, provides implementation scientists with a systematic approach for assessing contextual factors that influence PRO integration in clinical settings. Paired with our data collection forms (Supplementary Materials), this framework encompasses key observational domains, including physical environment, workflow patterns, staffing configurations, and implementation climate indicators, all of which proved valuable in characterizing the diverse implementation contexts across diverse CCC settings. We acknowledge that the training and experience of observers has a substantial impact on the quality of data collected. The open text box is designed to systematically capture cultural, relational and tacit operational dynamics. To further enrich the observation, structured reflection prompts for observers (e.g., how the observer’s positionality may have shaped what they noticed) may be included. While not consistently implemented in the study, periodic pauses during the observation period could also be built in for reflexive memoing.

**Table 4:** Field observation framework for pre-implementation of pre-patient-reported outcome measurement

**PRE-VISIT PREPARATION**
6–8 Weeks Before Site Visit
*** Stakeholder identification and engagement***
• Contact the site Principal Investigator (PI) and site champion to identify an appropriate administrative contact person
• Document contact information for all key implementation stakeholders
• Establish implementation timeline and communication expectations
• Begin documentation of site-specific contextual factors
*** Initial communication protocol***
• Establish a formal communication channel with the identified administrative contact
• Provide a comprehensive introduction to study aims and implementation objectives
• Articulate the purpose and scope of the site observation visit
• Schedule a preliminary virtual meeting (to occur no later than 4 weeks pre-visit)
• Send initial documentation package explaining site visit procedures
4–6 Weeks Before Visit
*** Institutional requirements assessment***
• Confirm all pre-visit health clearance requirements (e.g., vaccination records, TB testing, COVID protocols)
• Identify any required IT security documentation or clinic access protocols
• Document required observer forms and identity verification requirements
• Determine site credentialing needs (e.g., temporary badges, access cards)
• Document specific credentialing processes and locations
*** Logistical planning***
• Confirm precise clinic location, parking information, and security access details
• Map transportation routes between accommodations and implementation site
• Document public transportation options if applicable
• Establish contingency plans for travel disruptions (e.g., due to weather)
*** Contextual Information gathering***
• Document staffing patterns and anticipated personnel present during the site visit
• Identify optimal observation days based on patient volume and care delivery patterns
• Map weekly workflow variations to identify the highest-yield observation periods
• Document the clinic's least busy periods (to facilitate staff interviews, if applicable)
• Collect staff role descriptions, if available
*** Operational details***
• Exchange direct contact information for key personnel (cell phone numbers, email addresses)
• Document clinic operating hours, scheduled breaks, and lunch rotation schedules
• Confirm photography permissions and documentation restrictions for physical space
• Identify available meeting spaces for research team coordination during the visit
• Determine if specialized IT permissions are needed for on-site technology assessment
2–4 Weeks Before Visit
*** Site visit confirmation***
• Reconfirm visit details with the site champion, PI, and administrative contact
• Send a formal visit confirmation letter with a precise schedule and expectations
• Verify the completion status of all pre-visit documentation requirements
• Obtain written confirmation of visit parameters from site leadership
• Document any last-minute changes to clinic operations or staffing
*** Research team preparation***
• Confirm the number and roles of study staff attending the site visit
• Conduct pre-visit team briefing to review observation protocols
• Assign specific roles and observation responsibilities to team members
• Review data collection instruments and observation techniques
• Discuss site-specific contextual factors and areas of particular interest
*** Food and refreshment planning***
• Arrange food and refreshment delivery for clinic staff
• Consider dietary restrictions and preferences when possible
• Document delivery timing and location details
• Plan introductory remarks and project overview presentation (e.g., iPad or handouts to show the imPROVE platform)
1 Week Before Visit
*** Final confirmation***
• Send reminder emails to all site contacts
• Confirm food orders and delivery arrangements
• Review weather forecasts and adjust travel plans if necessary
• Conduct final team briefing to address any questions or concerns
• Verify travel arrangements and accommodations
*** Materials preparation***
• Prepare information packets about the research project for site staff
• Organize business cards and contact information for distribution
• Assemble all required documentation for site access
• Prepare backup copies of all essential forms and protocols
• Pack observation materials, recording devices, and backup supplies
**ON THE DAY OF THE VISIT**
Pre-clinic activities
*** Site access***
• Retrieve the required ID or visitor badge from the security
• Complete any same-day verification procedures
• Sign necessary confidentiality agreements if required
• Document badge return requirements or expiration dates
*** Early arrival***
• Arrive 15–20 min before the clinic opens
• Conduct brief team orientation to physical space
• Establish appropriate observation positions
• Test recording equipment if applicable
• Verify food delivery arrangements and timing
During clinic visit
*** Initial engagement***
• Introduce study team members to clinic staff
• Provide a concise overview of visit aims and purpose
• Explain observation methodology and non-disruptive approach
• Answer any questions from clinical staff
• Express appreciation for site participation
*** Environmental documentation***
• Capture photographs of clinic space (if permitted)
• Document clinic layout, workflow patterns, and physical arrangement
• Note the availability and placement of patient education materials
• Observe the placement of technology and potential PRO integration points
• Map patient flow through physical space
*** Professional protocol***
• Maintain non-interference with healthcare team huddles or clinical conferences
• Position observers strategically to minimize disruption
• Avoid patient care areas during sensitive discussions
• Follow all institutional regulations regarding observation
• Ensure that no protected health information is collected
*** Structured data collection***
• Utilize approved forms to document field observations – refine iteratively as needed
• Record timing of key workflow events (patient arrivals, transitions, provider interactions)
• Document observed barriers and facilitators to potential PRO implementation
• Note staff interactions, communication patterns, and workflow bottlenecks
• Document patient characteristics relevant to implementation (e.g., language diversity)
**POST-VISIT PROCEDURES**
Immediate activities
*** Data organization***
• Consolidate all field notes in the designated secure study folder
• Scan any paper documentation in electronic format
• Organize photographic documentation with appropriate labeling
• Create backup copies of all collected data
• Verify completeness of observation documentation
*** Confidentiality protection***
• Review all field notes for potentially identifying information
• Redact or transform sensitive details to protect confidentiality
• Apply consistent anonymization protocols to all documentation
• Verify compliance with IRB and institutional requirements
Post-visit analysis
*** Team debriefing***
• Conduct structured debriefing sessions with the research team
• Document initial impressions, key observations, and emergent themes
• Refine field observation data form as appropriate for subsequent visits
• Identify potential implementation barriers and facilitators
• Discuss methodological challenges and process improvements
• Compare observations with prior implementation site visits
*** Initial synthesis***
• Identify preliminary implementation strategies based on observations
• Document contextual variations that may influence implementation
• Begin mapping observed workflow to potential PRO integration points
• Identify site-specific adaptation needs for the intervention
Stakeholder engagement
*** Site feedback loop***
• Prepare a summary of field observations for site review
• Send structured observation documentation to the site team
• Request feedback on accuracy and completeness
• Document any clarifications or corrections from site personnel
• Express appreciation for site participation
*** Integration planning***
• Begin drafting site-specific implementation workflow based on observations
• (If appropriate) Identify necessary adaptations to PRO intervention
• Document technology integration requirements
• Develop preliminary training needs assessment
• Prepare for subsequent implementation phases

Our study has limitations.As with all observational analyses, this work has inherent limitations. Single-day observations employed in the study capture only a cross-sectional view and may miss cyclical or temporal variations in clinic operations. The potential for Hawthorne effects, i.e., altered behaviour due to observer presence, also warrants consideration [31, 32]. We attempted to mitigate this through “fly on the wall” positioning, minimal engagement during peak workflow periods, and the naturally high clinical volume that limited staff attention to observers. Nevertheless, the possibility of subtle behavioural modification cannot be discounted. Our focus on observable workflow elements may underestimate latent implementation determinants related to organizational culture or clinician attitudes toward PRO data [33, 34]. The in-depth staff and patient interviews will explore these determinants, thereby complementing the field observations. Finally, the question of whether pre-implementation contextual assessment measurably improves implementation outcomes must be situated within the existing research linking contextual determinants, strategy selection, and implementation success. Future work examining context-strategy-outcome fit across sites could identify which strategies are most effective under specific contextual conditions. This line of inquiry is critical for developing implementation strategies that optimize fit between context and intervention while enhancing sustainability and scalability across healthcare systems.

## Conclusion

This study advances implementation science by demonstrating how structured field observations can systematically document implementation determinants before PRO integration. The substantial contextual variation observed across CCCs underscores the importance of pre-implementation assessment in developing tailored, contextually responsive implementation strategies. Our methodological approach provides implementation scientists with a replicable framework for generating actionable insights, ultimately improving the precision and effectiveness of implementation efforts in complex healthcare settings.

## Supplementary Information

Supplementary Material 1.

## Data Availability

All data generated or analysed during this study are included in this published article [and its supplementary information files].

## Abbreviations

ACCC: Association of Cancer Care Centers
CCC: Community Cancer Center
CFIR: Consolidated Framework for Implementation Research
HIT: Health Information Technology
PRO: Patient-Reported Outcome
