# Supplementary material for: Field observation as a method to guide patient-reported outcome measurement integration in community cancer centers
Source: Implement Sci Commun. 2026 Jan 28;7:39. doi: 10.1186/s43058-026-00859-5 (PMC12924208; doi:10.1186/s43058-026-00859-5)
Supplement: Supplementary file 1 — Supplementary Material 1. [file 43058_2026_859_MOESM1_ESM.docx]

**SUPPLEMENTARY MATERIALS**

**CLINIC SPACE OBSERVATION LOG**

**Site name: ___________________________ Date: _______________**

**Observer: ___________________________**

**Clinic Hours: Start time: _________ End time: _________**

**Observation Period: Start time: _________ End time: _________**

**WAITING AREA ASSESSMENT**

***Physical Environment***

- Number of chairs available: _________
- Dedicated to breast cancer patients only? □ Yes □ No
- Space characteristics:
- Wi-Fi availability: □ Yes □ No □ Password required

***Available Materials in the Waiting Area***

**Posters/Flyers:** □ Yes □ No

- Topics: □ Research □ Clinical information □ Patient education □ Support services □ Other: ____________
- Languages available: □ English □ Spanish □ Other: ____________
- PRO-related content: □ Yes (describe: ____________) □ No

**Brochures/Handouts:** □ Yes □ No

- Topics: □ Research □ Clinical information □ Patient education □ Support services □ Other: ____________
- Languages available: □ English □ Spanish □ Other: ____________
- PRO-related content: □ Yes (describe: ____________) □ No

**Staff present vs. part of the team**

- Front desk staff: _____ vs _______ Scheduler: ________ vs ________
- Other staff: ______________: ________ vs _______

**PRO Implementation Potential**

- Suitable space for tablet/device use: □ Yes □ No □ Limited
- Potential staffing point for PRO assistance: □ Yes (position: ____________) □ No

**Additional Waiting Area Observations:**

**EXAMINATION AREA ASSESSMENT**

***Patient Flow***

- Intake hallway/area for vitals: □ Yes □ No
- The patient returns to waiting area after vitals: □ Yes □ No □ Sometimes
- PRO collection opportunity in this area: □ Yes □ No □ Limited

***Exam Room Environment***

Number of exam rooms available _________

Technology in room: □ Computer □ iPad □ Other: ____________

***Available Materials in Exam Rooms***

**Posters:** □ Yes □ No

- Topics: □ Research □ Clinical information □ Patient education □ Support services □ Other: ____________
- PRO-related content: □ Yes (describe: ____________) □ No

**Brochures/Handouts:** □ Yes □ No

- Topics: □ Research □ Clinical information □ Patient education □ Support services □ Other: ____________
- Available in multiple languages: □ Yes □ No
- PRO-related content: □ Yes (describe: ____________) □ No

**Staff present vs. part of the team**

- Breast cancer surgeon: _____ vs _______ Nurse practitioner: ________ vs ________
- Physician assistant: ______ vs ________ Medical Assistant: ________ vs _________
- Social worker: _______ vs __________ Nurse navigator: ________ vs __________
- Other staff: ______________: ________ vs _______

**STAFF WORKSPACE OBSERVATIONS**

Location of clinician workspaces: □ Dedicated area □ Exam rooms □ Both □ Other: ____________

Electronic health record access points: □ Workstations □ Portable devices □ Both

Potential PRO review locations: □ Provider workstations □ Exam rooms □ Both □ Other: ____________

**ADDITIONAL CLINIC OBSERVATIONS**

Document any potential PRO implementation opportunities, barriers, or other relevant observations not captured above:

1. **FRONT-END OBSERVATION LOG**

**Site name: ___________________________ Date: _______________**

**Observer: ___________________________**

**Clinic Hours: Start time: _________ End time: _________**

**Observation Period: Start time: _________ End time: _________**

| **Patient no.** | **Patient arrives at the clinic** | **Arrived with caregiver (Y/N)** | **Appointment type (New/Follow up)** | **Appointment time (if known)** | **Time patient checks in with the front desk** | **Patient called to the exam area** | **Patient returned to the waiting room before seeing a provider. (Y/N)** | **Patient returned from the exam area** | **Patient exits the clinic** |
| --- | --- | --- | --- | --- | --- | --- | --- | --- | --- |
| **1** |  |  |  |  |  |  |  |  |  |
| ***Notes*** |  | | | | | | | | |
| **2** |  |  |  |  |  |  |  |  |  |
| ***Notes*** |  | | | | | | | | |
| **3** |  |  |  |  |  |  |  |  |  |
| ***Notes*** |  | | | | | | | | |
| **4** |  |  |  |  |  |  |  |  |  |
| ***Notes*** |  | | | | | | | | |
| **5** |  |  |  |  |  |  |  |  |  |
| ***Notes*** |  | | | | | | | | |

**ADDITIONAL OBSERVATIONS**

Use this space to document overall impressions of the clinic workflow, potential PRO integration opportunities, workflow bottlenecks, or other implementation-relevant observations:

1. **BACK-END OBSERVATION LOG**

**Site name: ___________________________ Date: _______________**

**Observer: ___________________________**

**Clinic Hours: Start time: _________ End time: _________**

**Observation Period: Start time: _________ End time: _________**

| **Patient no.** | **Patient called to the exam area** | **Arrived with caregiver (Y/N)** | **Medical assistant (in-out time)** | **Physician Assistant (in-out time)** | **Nurse Practitioner (in-out time)** | **Physician (in-out time)** | **Interpreter used** | **Patient exits exam room** | **Follow-up scheduled (y/n) *if In the exam area** |
| --- | --- | --- | --- | --- | --- | --- | --- | --- | --- |
| **1** |  |  |  |  |  |  |  |  |  |
| ***Notes*** |  | | | | | | | | |
| **2** |  |  |  |  |  |  |  |  |  |
| ***Notes*** |  | | | | | | | | |
| **3** |  |  |  |  |  |  |  |  |  |
| ***Notes*** |  | | | | | | | | |
| **4** |  |  |  |  |  |  |  |  |  |
| ***Notes*** |  | | | | | | | | |
| **5** |  |  |  |  |  |  |  |  |  |
| ***Notes*** |  | | | | | | | | |

**ADDITIONAL OBSERVATIONS**

Use this space to document overall impressions of the clinic workflow, potential PRO integration opportunities, workflow bottlenecks, or other implementation-relevant observations:
